# P-181. Implementation of Chagas Disease Screening Programs in Cardiology Clinics within the Largest Safety Net Hospital System in the United States

**DOI:** 10.1093/ofid/ofaf695.405

**Published:** 2026-01-11

**Authors:** Karim J Gebran-Chedid, Leidy Valerio Perez, Maria Rosa Velasquez, Madeline DiLorenzo, Ellie Carmody, Masha Slavin, Eve Spear, Christina Coyle

**Affiliations:** New York Medical College - NYCHHC/Metropolitan, New York, NY; NYU Grossman School of Medicine - NYCHHC/Bellevue, New York, New York; New York Medical College - NYCHHC/Metropolitan, New York, NY; NYU Langone Health, New York, New York; NYU Langone Health, New York, New York; NYU Langone Health - NYCHHC/Bellevue, New York, New York; Albert Einstein College of Medicine - NYCHHC/Jacobi, New York, New York; Albert Einstein College of Medicine, Bronx, NY

## Abstract

**Background:**

Chagas disease (CD), caused by the parasite *Trypanosoma cruzi*, is endemic to Latin America. If left untreated, 20-30% of infected individuals will develop Chagas cardiomyopathy. We implemented a CD screening program across cardiology clinics within the New York City Health and Hospitals system, which serves a large at-risk patient population.

**Figure 1.**
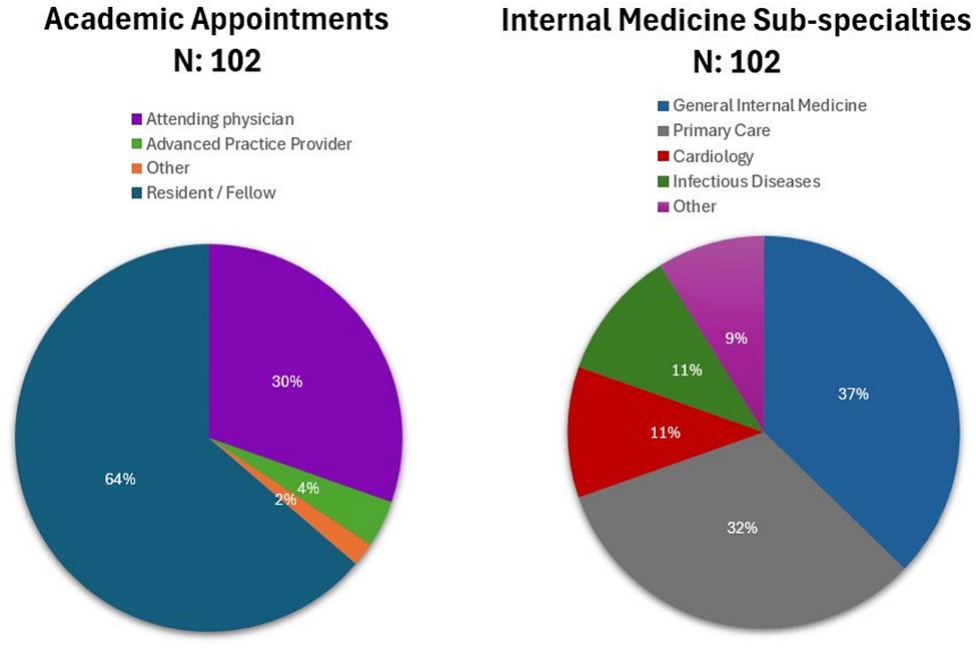
Characteristics of KAP Survey Respondents

**Figure 2.**
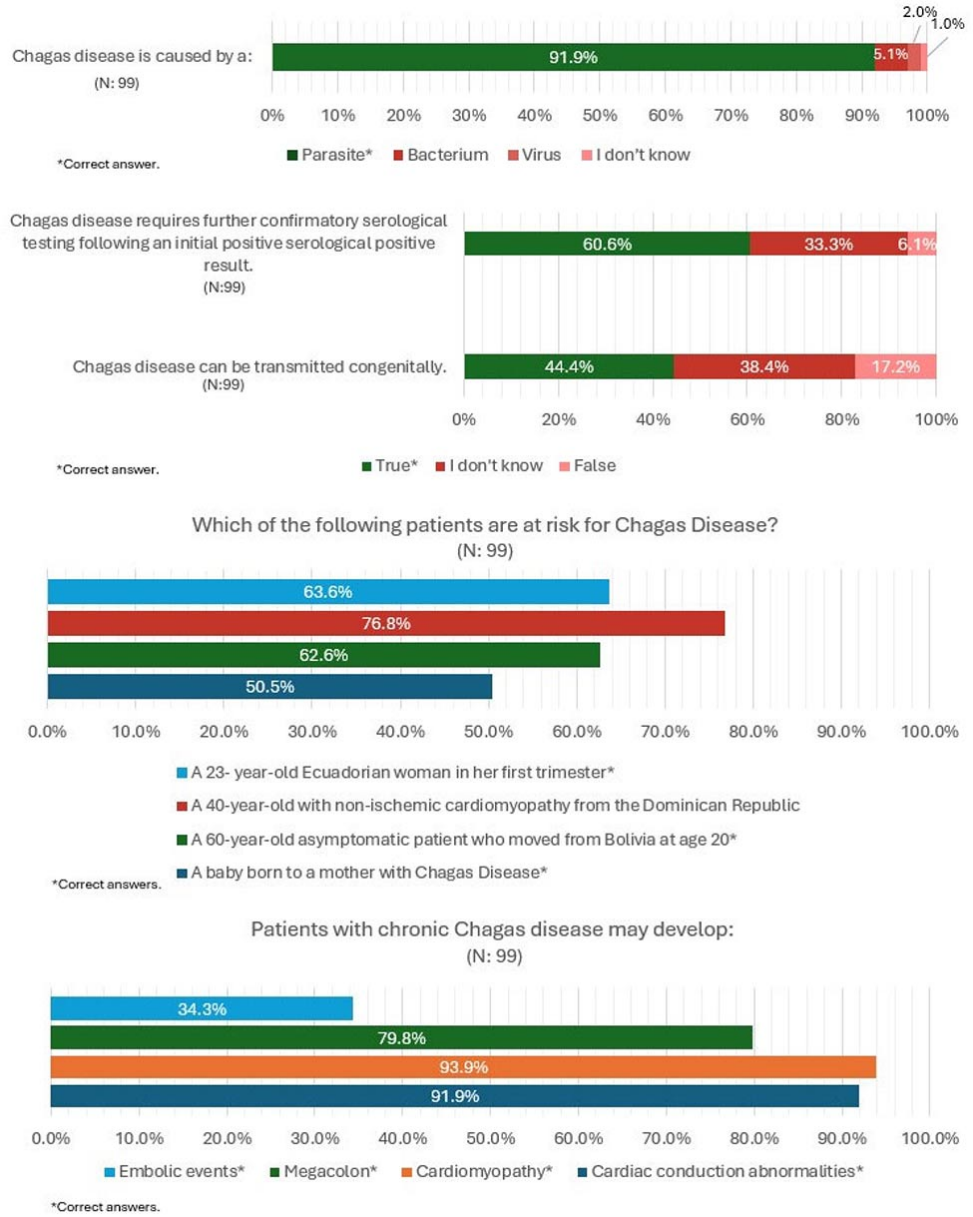
Selected Responses to Chagas Disease Knowledge Questions

**Methods:**

The program had three interventions: a provider knowledge, attitudes and practices (KAP) survey, provider educational sessions on CD, and implementation of risk-based serologic screening at three sites. Each site integrated a smartphrase into the electronic medical record prompting providers to identify at-risk patients, followed by targeted serologic testing. An implementation pilot was conducted at one site over the first year, which included monthly check-ins to address barriers.

**Figure 3.**
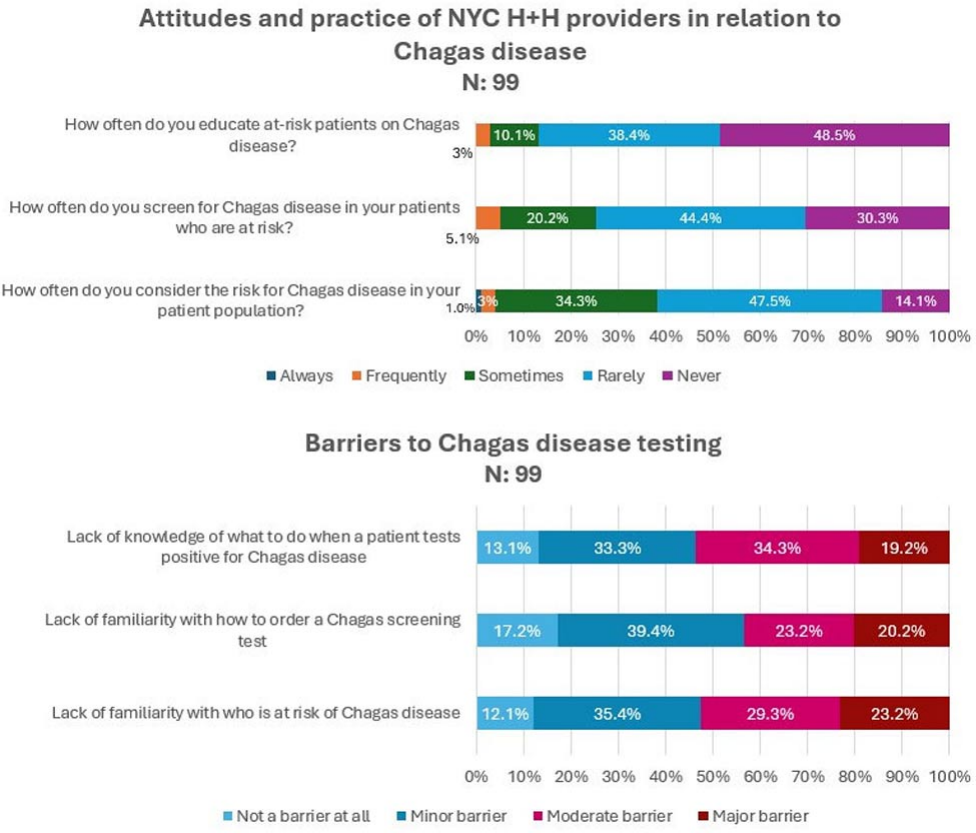
Attitudes, Practices and Barriers Related to Chagas Disease Screening

**Figure 4.**
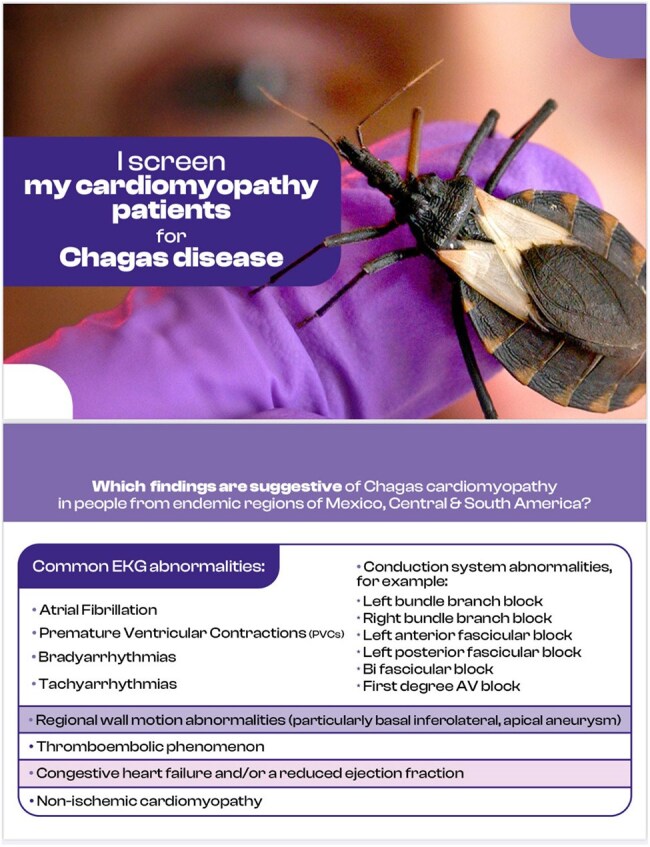
Educational Material: Chagas Disease and Cardiomyopathy Two-Face Reference Card.

**Results:**

Of 102 KAP survey respondents, 62% were residents. Most (88%) were not confident in their knowledge of CD, 77% incorrectly felt that the Dominican Republic was a country at risk, and over 50% were unaware of congenital transmission. While over 90% knew CD caused conduction abnormalities and cardiomyopathy, only 34% recognized that embolic events could develop. Over 12 months of screening at the pilot site, providers appropriately ordered tests in 231 (53%) eligible encounters with at-risk patients, and 128 (55%) of these patients completed initial testing. Four (3%) had a positive initial result and are pending confirmatory testing. In the 3 months prior to the pilot, no Chagas testing was performed.

**Conclusion:**

Our data highlight providers’ knowledge gaps regarding patients’ risk for CD and the extent of potential CD cardiac manifestations. After education, Chagas tests increased from none to over half of eligible encounters in this initial phase. Future efforts will focus on identifying challenges to provider adherence, improving workflow integration, and engaging patients to expand screening at all sites.

**Disclosures:**

All Authors: No reported disclosures

